# Diagnostic Value of Run Chart Analysis: Using Likelihood Ratios to Compare Run Chart Rules on Simulated Data Series

**DOI:** 10.1371/journal.pone.0121349

**Published:** 2015-03-23

**Authors:** Jacob Anhøj

**Affiliations:** Centre of Diagnostic Evaluation, Rigshospitalet, University of Copenhagen, Copenhagen, Denmark; Cardiff University, UNITED KINGDOM

## Abstract

Run charts are widely used in healthcare improvement, but there is little consensus on how to interpret them. The primary aim of this study was to evaluate and compare the diagnostic properties of different sets of run chart rules. A run chart is a line graph of a quality measure over time. The main purpose of the run chart is to detect process improvement or process degradation, which will turn up as non-random patterns in the distribution of data points around the median. Non-random variation may be identified by simple statistical tests including the presence of unusually long runs of data points on one side of the median or if the graph crosses the median unusually few times. However, there is no general agreement on what defines “unusually long” or “unusually few”. Other tests of questionable value are frequently used as well. Three sets of run chart rules (Anhoej, Perla, and Carey rules) have been published in peer reviewed healthcare journals, but these sets differ significantly in their sensitivity and specificity to non-random variation. In this study I investigate the diagnostic values expressed by likelihood ratios of three sets of run chart rules for detection of shifts in process performance using random data series. The study concludes that the Anhoej rules have good diagnostic properties and are superior to the Perla and the Carey rules.

## Introduction

Plotting data over time is a simple method to learn from trends, patterns, and variation in data over time and to study the effect of improvement efforts.

A run chart is a line graph of a quality measure over time with the median shown as a horizontal line dividing the data points so that half of the points are above the median and half are below.

The main purpose of the run chart is to detect process improvement or process degradation, which will turn up as non-random patterns in the distribution of data points around the median.

Run charts have many uses. Anything that can be expressed as counts or measures sampled over time can be analysed for non-random variation with run charts. In health care improvement, run charts are being used to monitor improvement in quality indicators—common examples being infection and complication rates, waiting times, readmissions, and adherence to standard procedures. Furthermore, run charts are useful for process control, that is, to monitor critical processes in order to detect process degradation quickly (1, 2, 3).

**Fig 1:**
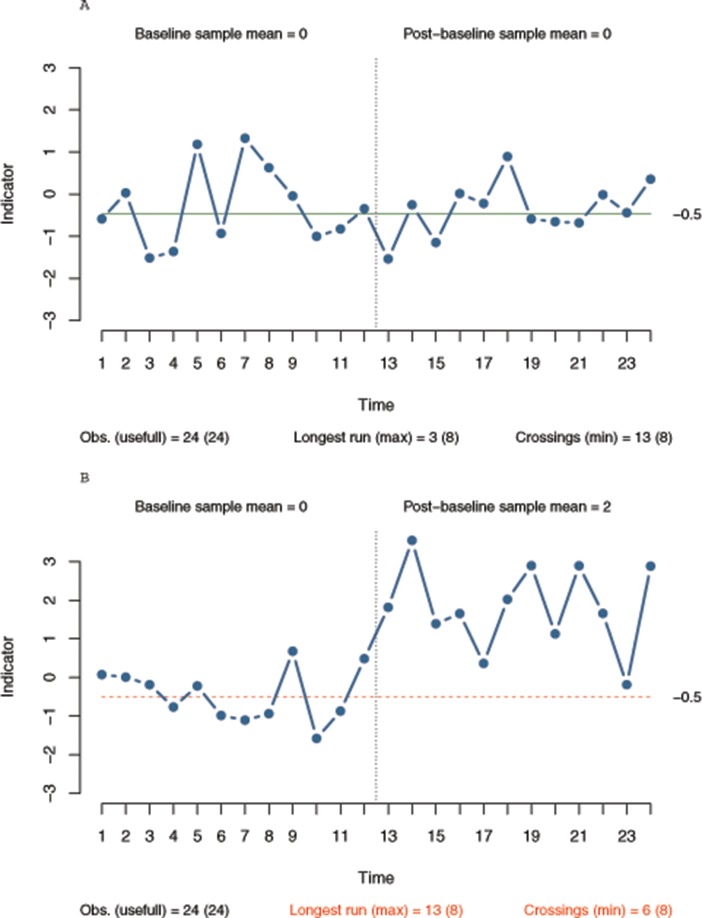
Example run charts with and without a shift in sample mean. Both charts have 24 useful observations, that is, data points not on the median. The median is calculated from the first 12 data points (baseline). **A**: No shift. The longest run has 3 data points, and the curve crosses the median 13 times. Only random variation is identified. **B**: A shift in sample mean of 2 SD was introduced in the last 12 data points. The longest run has 13 data points, which is above the signal limit of the Anhoej rules (8), and there are 6 crossing, which is below the signal limit (8), thus, non-random variation is identified. The plots were created with the qicharts package for R [4].

If the process of interest shows only random variation the data points will be randomly distributed around the median (Fig. 1A). Random meaning that we cannot know in advance if any single data point will fall above or below the median, but that the probability of each event is 50%, and that the data points are independent. Independence means that the position of one data point does not influence the position of the next data point, that is, data are not auto-correlated. If the process shifts or drifts, these conditions are no longer true and patterns of non-random variation may be detected by statistical tests.

Non-random variation may present itself in several ways. Especially, if the process centre is changing due to improvement or degradation we may observe “unusually” long runs of consecutive data points on the same side of the median or that the graph crosses the median “unusually” few times (Fig. 1B).

A run is defined as one or more consecutive data points on the same side of the median. Data points that fall on the median are not counted. They do neither contribute to the run nor do they break it. The distribution of runs and longest runs are fundamental to run chart analysis.

The expected number of runs was first studied by Swed and Eisenhart [5]. Recently, Chen proposed a simpler method counting crossings, which is the number of times the graph crosses the median [6]. Both methods are well modelled by statistical theory and yield comparable results. The general idea is that the number of crossings or runs in a random process is predictable within limits, and if there are more or less than predicted it is an indication that the process is not random.

Similarly, the length of the longest run is predictable within limits. However, there is no general agreement on what defines an “unusually long run”. Perla suggests runs of six or more data points to identify non-random variation [1], while Carey suggests seven or eight [2]. Based on the theory of long runs described by Schilling [7], we recently proposed a dynamic rule to identify too long runs [3]. According to this rule, the critical values depend on of the total number of data points in the run chart. Thus, in a run chart of 10 data points, a run of eight would be considered unusually long, while in a run chart of, say, 30 data points a run of eight would not.

Another commonly used test for non-random variation is the trend test, which identifies unusually long runs of data points all going up or down. While the trend test has been shown to be at best useless [3, 8], it is still widely used [1, 2].

It is common practice to bundle two or more run chart tests so that if just one of the tests is positive it is taken as suggestive evidence that non-random variation is present in the process of interest.

In this study I compare the diagnostic value of three sets of run chart rules that have been proposed in peer reviewed articles (Table 1).

**Table 1:** Three sets of run chart rules.

	Perla	Carey	Anhoej
Longest run	> = 6	> = 7 (8 if there are 20 or more data points in the run chart)	> UPL
Number of runs (or crossings)	< LPL or > UPL	< LPL or > UPL	< LPL
Longest trend	> = 5	> = 6	NA

UPL/LPL = dynamic upper/lower prediction limits based on the number of useful observations. See text for details.

The critical values for longest run and number of runs in Perla's and Carey's sets of rules are tabulated in Perla's paper [1].

The limits used with the Anhoej rules can be calculated using the formulas or looked up in a table provided in my previous paper [3]. The use of the Anhoej rules is demonstrated in Fig. 1.

The primary aim of this study was to evaluate and compare the diagnostic properties of different sets of run chart rules for detection of non-random variation in the form of shifts in process performance over time. Second, I wanted to suggest a method for future evaluation and study of run chart rules.

## Methods

### Likelihood ratios

Traditionally, likelihood ratios are used to evaluate how well clinical tests are able to discriminate between the presence and the absence of specific clinical conditions. In this study I applied likelihood ratios to simulated run charts, which may be considered diagnostic tests for non-random variation.

The questions of interest for run chart users are “what is the chance that a run chart with a positive runs test really contains non-random variation?” and “what is the chance that a run chart with a negative runs test really contains only random variation?”

Likelihood ratios are diagnostic measures designed to answer these kinds of questions [9]. Assume that a run chart tests positive for non-random variation. A perfect test would mean that the run chart would certainly come from a process with non-random variation (true positive, TP). However, some run charts with only random variation also test positive (false positive, FP). We therefore correct the true positive rate by the false positive rate by dividing one with the other. The positive likelihood ratio is defined as TP rate/FP rate = sensitivity/(1-specificity).

Likewise, if a run chart tests negative this could be a false negative (FN) rather than a true negative (TN). The negative likelihood ratio is defined as FN rate/TN rate = (1-sensitivity)/specificity.

A likelihood ratio greater than 1 speaks in favour of the condition being tested for, which in our case is non-random variation, while a likelihood ratio less than 1 speaks against non-random variation. The further a likelihood ratio is from 1, the more or less likely is the presence of non-random variation. As a rule of thumb, a positive likelihood ratio greater than 10 is considered strong evidence that the condition being tested for is present. A negative likelihood ratio smaller than 0.1 is considered strong evidence against the condition [10].

Thus, likelihood ratios allow us to quantify the probability of non-random variation in run charts and are useful quality characteristics of run chart rules.

### Run charts simulation

For the purpose of this study, I developed a simulation programme that automatically creates data series simulating run charts from random numbers from a normal distribution (or optionally, a poisson distribution) and applies the Perla, Carey, and Anhoej run chart rules to identify non-random variation. In half the simulations a shift in process mean of 2 standard deviations (SD) is introduced. So, for each simulated run chart the true presence or absence of a shift together with the presence or absence of signals from the runs analyses is known by the simulation programme allowing the programme to calculate likelihood ratios for each set of run chart rules.

For each simulated data series the median is calculated using the first 6, 12 or 18 data points as baseline. And the shifts (when present) are introduced in the post-baseline period of 6, 12 or 18 data points. Thus, there are nine combinations of baseline and post-baseline periods of different length allowing us to study the influence of these parameters on the diagnostic value of the tests.

For each of the nine possible combinations of baseline and post-baseline length, 1,000 simulations are performed with and 1,000 without post-baseline shift. In total, 18,000 run charts are simulated.

The simulation programme was built with R version 3.1.2 [11] with the add-on packages dplyr, tidyr, lattice, and latticeExtra. The programme code is available as supplementary material to this article (S1 File).

## Results

**Fig 2:**
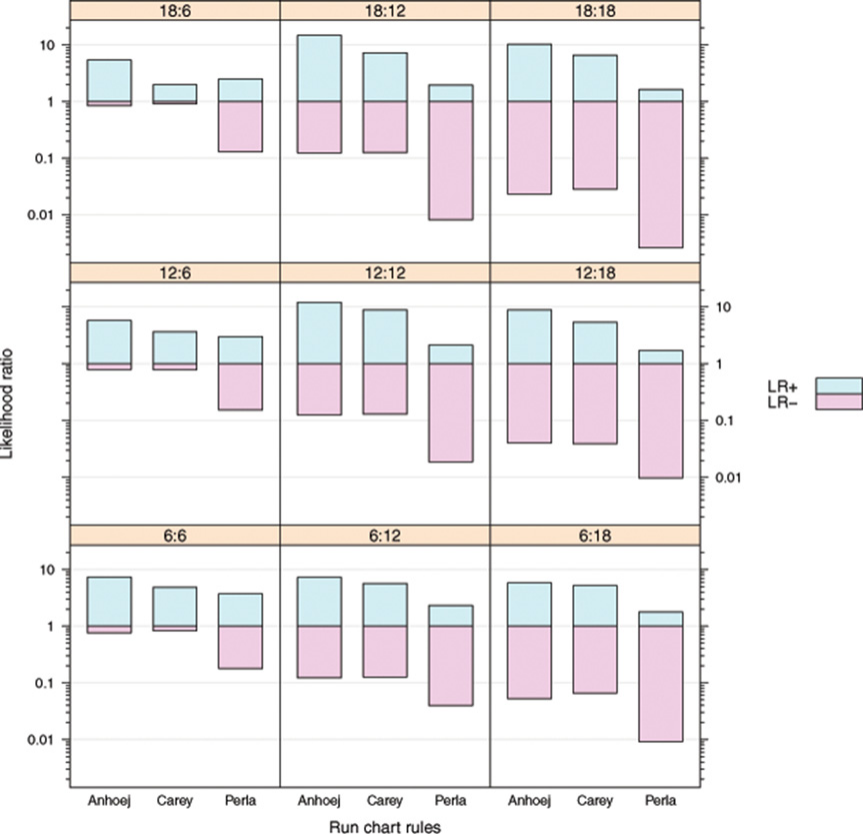
Likelihood ratios of run chart rules. The bars show the positive (LR+) and negative (LR-) likelihood ratios for each combination of run chart rules and baseline and post-baseline length shown in the panel header. Each panel is based on 2000 simulations of which 1000 had a shift of 2 SD in sample mean introduced in the post-baseline period.

Positive and negative likelihood ratios from the simulation study are displayed in Fig. 2. The length of baseline and post-baseline periods are shown in the panel headers. For example, the centre panel (12:12) is from 2000 run charts with 24 data points, 12 in the baseline period used for calculation of the median and 12 after a shift in sample mean of 2 SD was introduced in half of the run charts.

Table 2 shows the results from the 2000 simulations from the centre panel (12:12) of Fig. 2. Table 3 shows the calculations of likelihood ratios from the values in Table 2.

**Table 2:** Results from runs analyses of 2000 simulated run charts with 24 data points and a shift of 2 SD introduced in the last 12 samples of half the simulations.

	Anhoej		Carey		Perla	
	Shift -	Shift +	Shift -	Shift +	Shift -	Shift +
Signal -	927	115	901	116	534	10
Signal +	73	885	99	884	466	990

Shift +/- denotes the presence or absence of true shifts in process mean. Signal +/- denotes the result from the run chart analysis using the three set of run chart rules.

**Table 3:** Diagnostic properties of run chart rules based on the results from Table 2.

	Anhoej	Carey	Perla
Sensitivity TP/(TP+FN)	0.885	0.884	0.990
Specificity TN/(TN+FP)	0.927	0.901	0.534
LR+ sens/(1-spec)	12	8.9	2.1
LR- (1-sens)/spec	0.12	0.13	0.019

TP = true positive, FP = false positive, TN = true negative, FN = false negative, LR+/LR- = positive/negative likelihood ratio.

Comparable results were obtained using random data series from a poisson distribution (results not shown).

Overall, the Anhoej and Carey rules perform better than the Perla rules—the Anhoej rules slightly but consistently better than the Carey rules. For run charts with 12 or more data points in the post-baseline period, the Anhoej and Carey rules perform very well with positive LRs around 10 and negative LRs around 0.1. The interpretation is, that given a positive test based on the Anhoej or Carey rules, the presence of a shift of 2 SD is about 10 times more likely than no shift; and given a negative test, a shift is about 10 times less likely than no shift.

The Perla rules have very low negative LRs meaning that a negative test with great certainty rules out shifts. However, the Perla rules have rather low positive LRs suggesting that a positive test is only 2–4 times more likely to be associated with true shifts in process performance than not.

### A practical example

**Fig 3:**
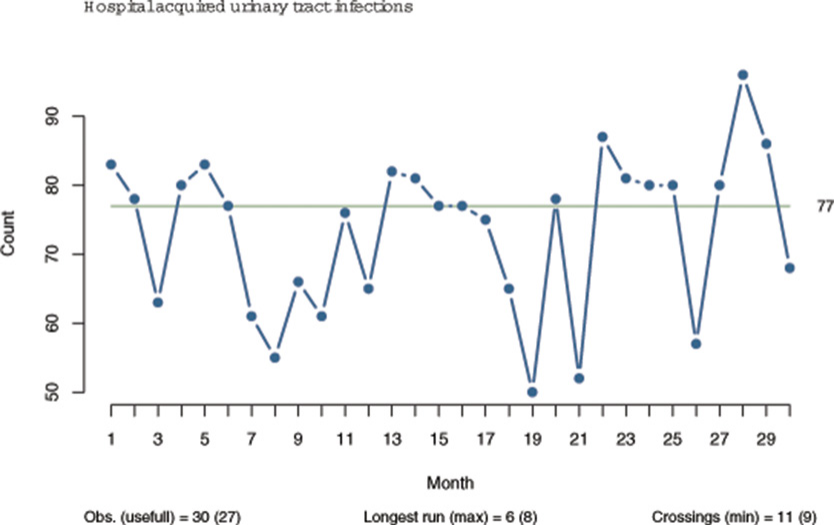
A practical example of false positive run chart analysis. The run charts displays the monthly number of hospital acquired urinary tract infection. The chart has 27 useful observations, the longest run is 6, and the number of crossings is 11. The longest run of data points going in the same direction (trend) is 6—points no. 13–19, since repeating values (15–16) only count as one. According to the Carey and Anhoej rules, the run chart shows only random variation. According to the Perla rules, the chart shows non-random variation in the form of a shift (longest run > 5) and a trend (longest trend > 4).

Fig. 3 displays the monthly number of hospital acquired urinary tract infection in an 800-bed acute care hospital in the Capital Region of Denmark. According to the Carey and Anhoej rules, the run chart shows only random variation. According to the Perla rules, the chart shows non-random variation in the form of a shift and a trend. Both signals have the desired direction (down), so one might conclude that improvement has occurred. However, data come from a period where no attempts were made to systematically target urinary tract infections at this hospital, and by looking at the chart as a whole, it is obvious that neither signal represents (persistent) improvement.

## Discussion

Run charts are widely used in health care improvement, but different sets of run charts rules exist, and few, if any, have critically evaluated the statistical properties of different rules. To my knowledge, this is the first study to characterise and compare the diagnostic properties of run charts. The results show that the Anhoej and Carey run chart rules have good diagnostic properties, while the Perla rules, due to a high number of false positive tests, are of questionable value.

As this study shows, the choice of run chart rules is of crucial importance when interpreting results from improvement programmes. Using very sensitive run chart rules will inevitably introduce a high risk of false positive tests. All other things being equal, sensitivity and specificity are opponents. The task is to find the best balance between the two. This balance is well expressed by likelihood ratios.

The availability of personal computers and free, open source software for data manipulation and analysis makes the use of simulations studies easily accessible to statistical researchers. But it is also important to realise that no number of cleverly designed simulations can cover the wide variety of real life conditions and situations. In this study I evaluated only nine carefully designed situations together with a fixed change in process performance. In practice, run chart rules are often applied dynamically while the process of interest is developing; and often baseline data are not available. In these situations, medians are recalculated and run chart rules applied after each new data point. Also, there are many more ways that change can appear than as a sudden and fixed shift in process performance at one specific point in time.

It is therefore important to stress that this study does not establish fixed diagnostic properties of run chart rules. Rather, it provides a framework for characterising and comparing run chart rules, while studying how different rules and conditions affect the conclusions from real life use of run charts.

“The proof of the pudding is in the eating.” It is my personal experience after having used the Anhoej rules on a wide variety of healthcare processes for several years that they work well in practice picking up significant changes in process performance while rarely creating false signals. However, more sensitive (and less specific) run chart rules may be appropriate in situations where it is important to identify changes very quickly or rule out non-random variation with high certainty, and where false positive signals are of minor importance. In any case, the choice of run chart rules should be decided before data collection begins.

As mentioned, the Anhoej rules perform slightly better than the Carey rules. This is mainly due to fewer false positive tests (73 vs. 99, Table 2). The difference may be explained partly by the different number of tests included in the two set of rules—the more tests performed, the more likely is a false positive result.

The Anhoej rules applies two tests to the run chart, while the Carey and Perla rules apply four tests including the trend test, which has been shown to be at best useless, and a test for unusually many runs, which is not included in the Anhoej rules. Unusually many runs (or crossings) is indeed a sign of non-random variation, which will appear if data are negatively auto-correlated, that is, if any high number tends to be followed by a low number and vice versa. However, this situation is rare in healthcare measures, and, if present, is most likely not an effect of improvement, rather than a result of poorly designed indicators or sampling issues. If, eventually, one wants to investigate whether data might be negatively auto-correlated, it would be useful also to include a test for unusually short longest runs.

Thus, the trend test and the test for too many runs are not useful for identifying shifts in process performance due to improvement, and adding them to the analysis will increase the risk of false positive tests and cause poorer run chart performance.

## Conclusions

In conclusion, this study shows that the Anhoej run chart rules have good diagnostic properties for identifying non-random variation in run charts under the conditions tested. Also the Anhoej rules are simpler to apply, because only two tests have to be performed, compared to the four tests included in the Carey and Perla rules. Finally, the Anhoej rules are independent of the number of available data points, making them useful also with run charts that have more than the usual 20–30 data points.

## Supporting Information

S1 File This file contains the R code to produce the simulations used in this study. The code will run on an installation of R with the add on packages lattice, dplyr, lattice, and latticeExtra. The output is a graph (Fig. 2) and a table showing likelihood ratios of run chart rules for identification of non-random variation in simulated run charts of different length with or without a shift in process mean.
(R) Click here for additional data file.
